# The regulatory role of m^6^A RNA methylation in insect sexual development: a review

**DOI:** 10.3389/fcell.2026.1802689

**Published:** 2026-04-29

**Authors:** Tianxiao Duan, Binbin Jin, Ye Xu, Jiabao Xu, Bin Cen

**Affiliations:** 1 Department of Quality Management, Hangzhou Center for Disease Control and Prevention (Hangzhou Health Supervision Institution), Hangzhou, Zhejiang, China; 2 Institute of Disinfection and Vector Control, Hangzhou Center for Disease Control and Prevention (Hangzhou Health Supervision Institution), Hangzhou, Zhejiang, China; 3 School of Basic Medical Sciences, Zhejiang Chinese Medical University, Hangzhou, Zhejiang, China

**Keywords:** alternative splicing, development, insect, N^6^-methylAdenosine (m^6^A), sex determination

## Abstract

N^6^-methyladenosine (m^6^A) is the most prevalent internal mRNA modification in eukaryotes and serves as a key post-transcriptional regulator in insect development. This review synthesizes current knowledge on m^6^A in insect sexual development, beginning with its well-established role in *Drosophila melanogaster*, where it ensures female fate by promoting the female-specific splicing of the master switch gene *Sex-lethal* (*Sxl*). Notably, classical female-lethal genes such as *Fl(2)d* and *Virilizer* are now recognized as essential regulatory subunits of the m^6^A methyltransferase complex, linking classic genetics to epitranscriptomics. In insects lacking an *Sxl*-centric pathway, such as Coleoptera, Hymenoptera, and Lepidoptera, m^6^A depletion does not disrupt core sex differentiation but instead plays essential roles in related processes, including metamorphosis in *Tribolium*, caste differentiation in honey bees, and gametogenesis across multiple orders.These findings underscore that m^6^A functions extend beyond the initial sex determination switch to regulate a broad spectrum of sex-related developmental processes across insect taxa, providing insights into the evolution of epigenetic regulation.

## 1 Introduction

Insect sex determination represents a key subject in developmental and evolutionary biology, with diverse mechanisms employed across taxa. These mechanisms include genetic factors such as autosome-to-sex chromosome ratio and recessive lethality, haplodiploidy—as observed in Hymenopteran *bees* where males are haploid and females diploid (Kageyama et al., 2012; Laslo et al., 2023), and environmental influences like nutritional cues (Bi et al., 2022; Wang et al., 2024). Previous studies have established that insect sex determination frequently relies on the alternative splicing of critical transcription factors to produce functional, sex-specific proteins. The foundational role of *Sxl* as the master regulator of sex determination was first demonstrated through genetic analyses showing that its activity is required for female development (Cline, 1988; Bell et al., 1988). Subsequent studies revealed that *Sxl* regulates the alternative splicing of Tra pre-mRNA, leading to the production of functional TRA protein only in females (Boggs et al., 1987; Nagoshi et al., 1988). TRA, together with the constitutive factor TRA2, directs the female-specific splicing of *Dsx* pre-mRNA, whereas in males, the absence of functional TRA results in the default male-specific splicing pattern (Burtis and Baker, 1989; Hoshijima et al., 1991). These molecular events collectively ensure the establishment and maintenance of sexual identity in *Drosophila* (Verhulst et al., 2010; Suzuki, 2018). While these transcriptional and splicing regulatory networks are well-established, the emerging field of epitranscriptomics raises a new question: how do RNA modifications, such as methylation, fine-tune or even determine sexual fate at the post-transcriptional level? N^6^-methyladenosine (m^6^A) RNA methylation, a key mechanism of such post-transcriptional regulation, has recently been discovered to be intimately linked to insect sex differentiation and development(Lence et al., 2016; Haussmann et al., 2016; Kan et al., 2017; Guo et al., 2018; Binta et al., 2023). This review aims to comprehensively synthesize the mechanistic roles and research advances concerning m^6^A methylation in insect sex determination, encompassing all major insect orders. It will cover molecular mechanisms, regulation of sex determination gene pathways, investigative methodologies, spatiotemporal dynamics during development, environmental influences, and similarities and differences across taxa.

## 2 Mechanisms and research methodologies for m^6^A modification

**FIGURE 1:**
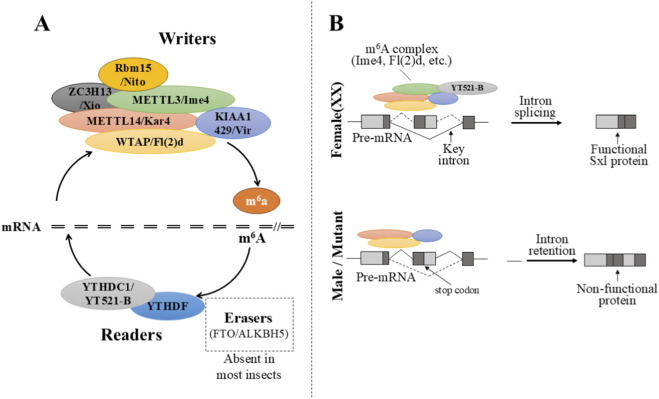
The core m^6^A machinery in insects and its regulation of *Sxl* splicing in Drosophila. **(A)** Schematic of the conserved insect m^6^A methyltransferase complex. The “Writer” proteins (METTL3/*Ime4*, METTL14/*Kar4*, WTAP/*Fl(2)d*, *Virilizer*) deposit m^6^A on target mRNAs, which is recognized by “Reader” proteins (YTHDC1/YT521-B, YTHDF) to influence RNA metabolism. Known “Eraser” enzymes, such as FTO/ALKBH5, are typically absent in insects. **(B)** Model of m^6^A-dependent alternative splicing of the *Sxl* pre-mRNA. In females (XX), the m^6^A complex modifies a key intron, recruiting YT521-B to promote its excision and the production of functional SXL protein. In males or m^6^A-deficient backgrounds, the intron is retained, introducing a premature termination codon that leads to a non-functional protein, directing male development or lethality.

m^6^A modification is governed by dedicated “writer”, “eraser”, and “reader” factors (Figure1A), whose dynamic reversibility permits precise regulation of mRNA function (Liu et al., 2014; Huang et al., 2020). The core m^6^A writer complex consists of a METTL3-METTL14 heterodimer, which relies on auxiliary factors, including WTAP, VIRMA/Virilizer, RBM15 and ZC3H13, for proper localization and catalytic activity (Liu et al., 2014; Ping et al., 2014; Yue et al., 2018). Insects possess conserved homologs of these key factors, exemplified by *Drosophila Ime4* (*dMETTL3*), *Kar4* (*dMETTL14*), *Fl(2)d* (*dWTAP*), and *Virilizer* (*dKIAA1429*) (Haussmann et al., 2016; Kan et al., 2017). Notably, these genes were originally identified through classical genetic screens as essential for female sex determination in fruit flies long before their molecular function in RNA methylation was understood. For instance, *Fl(2)d* and *virilizer* were reported in the 1990s as factors required for the female-specific splicing of *Sxl* (Granadino et al., 1990; Hilfiker et al., 1995). Mutations in these genes caused female-specific lethality and disrupted the production of functional SXL protein, yet their biochemical roles remained elusive for decades. It was only with the discovery of the m^6^A methyltransferase complex that these proteins were recognized as core components—*Fl(2)d* as the *Drosophila* homolog of WTAP, and *Virilizer* as the homolog of KIAA1429/VIRMA—thereby establishing a direct molecular link between epitranscriptomic modification and sex determination (Lence et al., 2016; Kan et al., 2017; Knuckles et al., 2018).

It is worth noting, however, that genetic analyses in *Drosophila* reveal a phenotypic divergence between mutations affecting different components of the m^6^A machinery. Null alleles of the catalytic subunits *Ime4* and *Kar4* are homozygous viable, albeit with developmental defects, whereas mutations in the auxiliary factors *Virilizer* and *Fl(2)d* are lethal (Haussmann et al., 2016; Lence et al., 2016). This discrepancy does not necessarily imply m^6^A-independent functions for *Virilizer* and *Fl(2)d*, as no such activities have been demonstrated to date. Instead, it may reflect the critical requirement for these auxiliary factors in facilitating m^6^A deposition on a specific subset of targets—such as *Sxl* during early embryogenesis—where the catalytic core alone is insufficient to ensure proper modification. This interpretation aligns with the established role of auxiliary factors in substrate recognition and complex stabilization (Knuckles et al., 2018), and underscores the importance of distinguishing between the catalytic core and essential regulatory components of the m^6^A writer complex when interpreting mutant phenotypes. This historical trajectory illustrates how classical developmental genetics and modern epitranscriptomics converged to reveal the mechanistic basis of a long-standing biological puzzle.

m^6^A “reader” proteins primarily belong to the YTH domain-containing family, which recognize and bind m^6^A-modified RNA to mediate functional outcomes (Kan et al., 2017; Lence et al., 2019). In contrast to mammals encoding multiple YTHDF paralogs, insect genomes generally contain fewer YTH domain proteins (Haussmann et al., 2016; Kan et al., 2017). For instance, *Drosophila* possesses only two YTH readers (nuclear YT521-B and cytoplasmic Ythdf), and this limited number of YTH domain-containing proteins is also observed in other insects such as honey bees (Hongay and Orr-Weaver, 2011; Horiuchi et al., 2013). This indicates that the insect m^6^A pathway likely operates through a smaller and simpler set of readers compared to mammals, which may reflect a more streamlined regulatory network for mediating m^6^A functions (Haussmann et al., 2016).

For demethylation (“erasure”), enzymes FTO and ALKBH5 have been identified in mammals and other vertebrates as capable of removing m^6^A modifications(Huang et al., 2020). Early bioinformatic analyses suggested that insects lack clear homologs of the canonical mammalian m^6^A demethylases FTO and ALKBH5 (Figure 1A). This led to a model where m^6^A regulation in insects might rely more on dynamic writing and reader-mediated decay rather than active erasure. However, a more complex and taxonomically variable picture has recently emerged. A comprehensive search across insect genomes revealed that while ALKBH5 homologs are present in some orders—including termites, beetles, and true bugs—they are absent in representative dipteran and lepidopteran species (Dai and Asgari, 2023). More importantly, functional evidence now points to alternative erasers. Experimental studies have demonstrated that ALKBH8 can reduce m^6^A levels on *Aedes aegypti* and *D. melanogaster* RNAs, suggesting it serves as a candidate m^6^A eraser in insects (Dai and Asgari, 2023). Collectively, these findings indicate that the presence and identity of m^6^A erasers in insects are more diverse than previously appreciated, with ALKBH8 potentially fulfilling this role in an order-specific manner. Collectively, the core machinery of the m^6^A modification system is broadly conserved across insects (Haussmann et al., 2016). Systematic analysis of 207 insect genomes demonstrates high evolutionary conservation of m^6^A-associated genes. Their subcellular localization patterns also mirror those in mammals: for instance, in silkworms, BmMETTL3/14 and YTHDC are nuclear, while YTHDF is cytoplasmic (Haussmann et al., 2016). This conservation provides a solid foundation for investigating m^6^A functions using insect models.

Researchers have utilized diverse emerging methodologies to elucidate m^6^A functions in insects. MeRIP-seq (m^6^A RNA Immunoprecipitation followed by sequencing) is a widely used technique for transcriptome-wide mapping of m^6^A modifications. This method employs m^6^A-specific antibodies to immunoprecipitate methylated RNA fragments, which are then subjected to high-throughput sequencing (Liu et al., 2014; Wang et al., 2018). To date, transcriptome-wide m^6^A profiling has been performed in several insect species, including *D*. *melanogaster*, *Anopheles sinensis*, *Bombyx mori*, *Tribolium castaneum*, and *Apis mellifera* (Lence et al., 2016; Li et al., 2019; Wang et al., 2021; Liu et al., 2022; Jiao and Palli, 2023). Comparative analyses reveal both conserved and species-specific features: while m^6^A enrichment near stop codons and in 3′ UTRs is broadly conserved across eukaryotes, insects exhibit unique patterns such as the pronounced m^6^A deposition within coding regions observed in *An. sinensis* and the stage-specific methylation dynamics during *B. mori* development (Li et al., 2019; Liu et al., 2022). These findings highlight the evolutionary plasticity of m^6^A distribution and its potential adaptation to diverse insect life histories.

CLIP (UV Crosslinking and Immunoprecipitation) techniques have also been employed to pinpoint the interaction sites between m^6^A reader proteins and their target RNAs (Chen et al., 2015b; Liu et al., 2022). This allows for the identification of specific binding sites of YTH family proteins on m^6^A-modified mRNAs, facilitating the association of particular modifications with changes in splicing or stability (Wang et al., 2014). For functional validation, CRISPR/Cas9-mediated gene knockout and RNA interference (RNAi) serve as principal tools in insects for elucidating m^6^A functions. CRISPR/Cas9 is employed in model insects like *Drosophila* to generate knockouts of genes such as “*METTL3*/*METTL14*”, enabling observation of mutant sex phenotypes and molecular alterations (Batista et al., 2014; Geula et al., 2015). Conversely, RNAi is extensively used in insects less tractable for genetic manipulation, such as the red flour beetle *T*. *castaneum* and the silkworm *B. mori*, achieving efficient target gene silencing via dsRNA injection. For example, RNAi knockdown of multiple m^6^A writers and readers in *T*. *castaneum* demonstrated that silencing the core writer enzyme results in phenotypes including molting defects during larval-pupal ecdysis and adult sterility (Chen et al., 2015a).

Additionally, spatiotemporal transcriptomic analysis represents a key strategy for uncovering m^6^A functions (König et al., 2010; Van Nostrand et al., 2016; Xia et al., 2024). This involves assessing the expression dynamics of m^6^A machinery components and target genes, or m^6^A modification levels themselves, across different developmental stages and tissues. For instance, integrating single-cell transcriptomics with MeRIP-seq (scMeRIP-seq) enables the exploration of m^6^A dynamics within specific cell lineages. Alternatively, time-course RNA sequencing can compare transcriptional profiles in the presence or absence of m^6^A modifications during key sex determination windows, such as early embryogenesis (Ule et al., 2003). The combined application of these methodologies allows for a multi-faceted and multi-layered elucidation of the mechanisms by which m^6^A regulates insect sex determination (Meyer et al., 2012; Chen et al., 2015a; Xia et al., 2024).

## 3 Background of sex determination

Insects exhibit diverse sex determination mechanisms, yet they universally converge on genetic pathways that regulate the expression of downstream sex differentiation genes (Sawanth et al., 2016). In Diptera, such as Drosophilidae *fruit flies*, the classical sex determination pathway is initiated by the X chromosome-to-autosome (X:A) ratio. Zygotes with a high X:A ratio (XX females) activate the expression of the master switch gene *Sxl*, producing functional maternal-type SXL protein. In contrast, zygotes with a low ratio (XY males) fails to activate *Sxl* (Hudry, 2025). In females, SXL protein, acting as the master regulator, maintains its own expression through autoregulatory alternative splicing and acts on the precursor mRNA of *Transformer* (*Tra*), promoting its splicing to generate functional TRA protein (Hudry, 2025). *Tra*, in concert with the cofactor *Tra2*, directs the alternative splicing of the downstream *Doublesex* (*Dsx*) precursor mRNA into the female-specific isoform (*Dsx* ^F^), thereby initiating the female developmental program (Hudry, 2025). Conversely, in males, due to the absence of *Sxl* and *Tra* activity, *Dsx* is constitutively spliced into the male isoform (*Dsx* ^M^), directing male development (Hudry, 2025) (Figure 1B).

Many non-dipteran insects do not utilize *Sxl* as the sex switch; instead, *Tra* or analogous factors serve this role directly (Verhulst et al., 2010). For instance, in *Hymenopteran bees*, the *Complementary sex determiner* (*Csd*) gene in heterozygous females produces active protein, initiating the female-specific splicing of *fem* (the *Tra* homolog). Diploid female bees generate FEM^F^ protein via this pathway, subsequently leading to female-specific splicing of *Dsx*, while haploid males, lacking a functional *csd* allele, produce only DSX^M^ (Mohr et al., 2017). Lepidopteran insects, including moths and butterflies such as the silkworm *B*. *mori*, employ a distinct sex determination mechanism. In this system, female development is typically determined by a female-specific W chromosome factor. For example, in *B. mori*, a female-specific piRNA silences the male-determining gene *Masc*, thereby permitting female development (Sawanth et al., 2016). Downstream of this initial signal, sexual dimorphism is similarly realized through the conserved *Tra*/*Dsx* cascade (Sawanth et al., 2016).

Some insects are also influenced by environmental factors. For example, nutrition and colony conditions in social insects like bees determine whether an individual develops into a reproductive queen or a sterile worker (despite both being genetically female, their developmental fates differ significantly) (Sawanth et al., 2016). In certain extreme conditions, environmental temperature can influence offspring sex ratio or phenotype (Sawanth et al., 2016). Regardless of the specific pathway, insect sex determination universally involves the hierarchical regulation of a cascade of sex-related genes, where post-transcriptional regulation—particularly alternative splicing and mRNA stability control—represents a critical node in determining sexual fate (Coelho and Smith, 2014; Hudry, 2025). Precisely because of this, RNA modifications like m^6^A have the potential to intervene in sex determination pathways by influencing the splicing, transport, or translation efficiency of mRNAs encoding key sex-determining factors, an emerging concept supported by initial findings in model insects (Wen et al., 2024).

## 4 Regulatory roles of m^6^A in insect sex determination pathways

The model organism *D*. *melanogaster* represents the most comprehensively studied insect species in the context of the relationship between m^6^A and sex determination (Lence et al., 2016). Several key studies have demonstrated that the m^6^A pathway is essential for female-specific alternative splicing of the *Sxl* gene, which initiates female development (Lence et al., 2016). Notably, Lence et al. (2016) showed that loss-of-function mutations in core m^6^A methyltransferase complex components, such as *Ime4*, lead to severe female-specific developmental defects, with mutant females producing aberrant *Sxl* mRNA isoforms resembling those of males. However, the phenotypic consequences differ depending on which components are affected. In *Drosophila*, null mutations in the catalytic subunits *Ime4* or *Kar4* cause female-specific *Sxl* splicing defects primarily in the brain, but do not produce visible female-to-male transformations in other somatic tissues such as genitalia or sex combs (Kan et al., 2017). Surviving mutant females exhibit behavioral and neurological defects rather than overt sexual transformations. In contrast, more severe masculinization—including development of male genitalia and sex combs in genetic females—occurs only when catalytic subunit mutations are combined with loss-of-function alleles of auxiliary factors such as *Fl(2)d* or *Virilizer* (Kan et al., 2017; Lence et al., 2016). This differential requirement suggests tissue-specific roles for the m^6^A machinery. The catalytic core is broadly required for m^6^A deposition, auxiliary factors may be particularly critical for modifying key targets in tissues where precise splicing regulation is essential for sexual differentiation.

**FIGURE 2:**
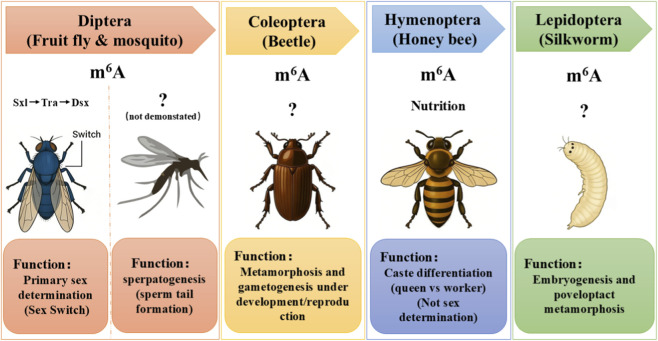
Divergent roles of m^6^A in insect development across taxa. This schematic compares the functions of m^6^A in representative species from four insect orders. In Diptera (*fruit fly*), m^6^A acts as a primary sex determination switch, regulating the *Sxl*-*Tra*-*Dsx* splicing cascade. In Diptera (genus *Anopheles*), m^6^a plays a role in mosquito spermatogenesis. In other orders, m^6^A governs broader developmental processes: Coleoptera (beetle) in metamorphosis and gametogenesis; Hymenoptera (honey bee) in nutrition-mediated caste differentiation; and Lepidoptera (silkworm) in embryogenesis and metamorphosis.

Building on this, Haussmann et al. (2016) provided mechanistic insight by demonstrating that m^6^A promotes the exclusion of a sex-specifically retained intron in the *Sxl* pre-mRNA (Haussmann et al., 2016). In *Ime4*-deficient genetic females, this intron is retained, resulting in non-functional *Sxl* transcripts and failure to activate downstream *Tra* and *Dsx* pathways, producing “pseudomale” phenotypes (Haussmann et al., 2016). The majority of XX individuals with disrupted m^6^A signaling undergo developmental arrest or perish due to the absence of functional SXL protein (Haussmann et al., 2016). Crucially, the m^6^A reader protein YT521-B (also known as dYTHDC1) plays a central role in interpreting these epitranscriptomic marks (Lence et al., 2017). Loss of YT521-B mirrors the phenotype of *Ime4* mutants, confirming its role in facilitating correct *Sxl* splicing via recognition of m^6^A marks on the pre-mRNA (Lence et al., 2017). This protein likely recruits splicing regulators or stabilizes specific RNA conformations that enable female exon inclusion and intron removal (Lence et al., 2017). In addition to its role in *Sxl* regulation, m^6^A also influences dosage compensation by regulating *msl-2*, a gene normally repressed by *Sxl* in females. In m^6^A-deficient XX embryos, *msl-2* is misexpressed, potentially disrupting X-chromosome dosage equilibrium (Wang et al., 2014). Beyond *Sxl*, genome-wide transcriptomic analyses reveal that m^6^A impacts the splicing and stability of numerous other mRNAs, including those involved in neural development and metabolism, although none are as sex-determination-critical as *Sxl* (Soldano et al., 2020). This link is further solidified by the fact that classical female-lethal genes like *Virilizer* and *Fl(2)d*, originally identified for their role in *Sxl* splicing (Granadino et al., 1990; Hilfiker et al., 1995), are now known to encode core components of the m^6^A methyltransferase complex (see Section 2). Collectively, current research establishes a robust model wherein m^6^A modification and its reader proteins act as post-transcriptional gatekeepers of female development, ensuring proper exon choice in the *Sxl* pre-mRNA and thus enabling sex-specific fate decisions in *Drosophila* (Lence et al., 2016; Haussmann et al., 2016) (Figure 2).

Despite this progress, the precise molecular mechanism by which YT521-B promotes female-specific *Sxl* splicing, and the basis for the phenotypic differences between catalytic and auxiliary subunit mutants, remain to be fully elucidated (Zhu et al., 2023). Furthermore, the phenotypic differences between catalytic and auxiliary subunit mutants warrant further investigation. Understanding whether these differences arise from differential target specificity, temporal requirements, or as-yet-unidentified m^6^A-independent functions will require the development of separation-of-function alleles and tissue-specific rescue experiments.

In contrast to *Drosophila*, no such direct impact of m^6^A on sex determination has yet been discovered in many other insects. This discrepancy correlates with the distinct sex determination strategies employed by different species. For example, Coleoptera insects like the red flour beetle (*T*. *castaneum*) do not utilize *Sxl* as a molecular sex switch, relying primarily on the *Tra*/*Dsx* pathway. Studies in *Tribolium* show that knockdown of its m^6^A writer enzymes, such as TcMETTL3, does not affect sex differentiation. No imbalance in sex ratio or discernible changes in male/female phenotypes were observed in experiments. Furthermore, m^6^A-deficient beetles developed normally into morphologically distinct males and females (Jiao and Palli, 2023). Further molecular analysis confirmed that the mRNA splicing of *Tra*, *Tra2*, and *Dsx* genes remained unaltered in treated beetles (Jiao and Palli, 2024), supporting the conclusion that m^6^A is not a required regulatory layer for their sex determination (Figure 2).

However, this does not imply that m^6^A is unimportant for beetle development; on the contrary, m^6^A is crucial for other processes. For instance, RNAi targeting key m^6^A pathway genes in late-instar Tribolium larvae causes ecdysis failure during adult emergence (Jiao and Palli, 2023), even though adult morphology develops normally. Furthermore, m^6^A deficiency severely impacts reproductive development: RNAi males exhibit underdeveloped testes and defective gametes, while females show drastically reduced egg number and size, and even laid eggs arrest development early in embryogenesis (Jiao and Palli, 2023; Zhang et al., 2025). These findings highlight that m^6^A in these species acts as an essential regulator of germline development and gametogenesis rather than a primary sex-determining switch. While m^6^A is dispensable for sex determination in *Tribolium*, its essential roles in metamorphosis and gametogenesis remain mechanistically unclear. Key questions include which specific m^6^A targets mediate ecdysis failure and whether m^6^A readers function differently in somatic versus germline tissues. Current RNAi-based evidence may not achieve complete depletion, and the lack of stable mutant lines limits assessment of m^6^A functions across generations.

In Hymenopteran bees, represented by the honey bee (*Ap*. *mellifera*), social insects possess a unique haplodiploid sex determination mechanism strongly influenced by environmental feeding, such as royal jelly. To date, no evidence suggests m^6^A directly participates in the *Csd*-*Fem*-*Dsx*-mediated sex differentiation in bees. However, m^6^A has been discovered to significantly influence the environmentally dependent developmental decision of queen versus worker differentiation. Worker larvae exhibit globally higher m^6^A levels on mRNA compared to same-aged queen larvae, and chemically inhibiting m^6^A levels in larvae destined to become workers induces partial queen phenotypes, such as ovary development (Wang et al., 2021). Studies in adult bees also provide evidence that m^6^A regulatory genes are differentially expressed across developmental stages and tissues, and may play a role in maintaining the physiological differences between reproductive and non-reproductive individuals. For example, expression of m^6^A methyltransferase genes—such as METTL3—and global m^6^A levels vary during larval development and are correlated with caste-specific developmental outcomes (Wang et al., 2021). These findings suggest that m^6^A modification in insects not only influences sex differentiation but may also mediate complex phenotypes such as caste identity and reproductive potential (Figure 2). The causal relationship between m^6^A levels and caste fate remains unresolved. While Wang et al. (2021) showed that m^6^A inhibition induces queen-like features, conflicting Nanopore sequencing data found few caste-specific m^6^A differences and no correlation with transcript expression. These contradictory results highlight the need for methodological standardization and functional validation. This role in nutrition-mediated caste differentiation represents a unique functional adaptation of m^6^A in social Hymenoptera, distinct from its function as a primary sex determination switch in *Drosophila* or its role in metamorphosis in *Tribolium*.

For Lepidoptera insects, exemplified by the silkworm (*B*. *mori*), no current evidence indicates direct involvement of m^6^A in its ZW-type sex determination pathway. However, m^6^A modification has been demonstrated to play a vital role in post-embryonic development, especially during metamorphosis. For example, knockdown of *Fl(2)d*—a key component of the m^6^A writer complex—leads to severe defects in larval-pupal transitions, indicating that proper m^6^A methylation is essential for developmental progression (Huang et al., 2023). While the function of METTL3 and YTHDF proteins during embryogenesis remains to be determined, the spatiotemporal expression of m^6^A-related genes across developmental stages suggests precise temporal and tissue-specific regulation (Figure 2). Although Liu et al. (2023) identified JH-regulated m^6^A targets including BmSPI4 and BmSPI5, the full repertoire of m^6^A target genes mediating embryonic development and silk gland function remains unknown. Recent studies revealed additional m^6^A roles in antiviral defense and diapause regulation, but how these functions intersect with sex determination remains unexplored. Genetic loss-of-function tools in silkworm remain limited compared to *Drosophila*. Thus, in Lepidoptera, m^6^A has been co-opted to support post-embryonic developmental programs and tissue-specific functions like silk gland regulation, further illustrating the evolutionary plasticity of this modification across insect orders.

In mosquitoes of the genus *Anopheles*, emerging transcriptomic studies reveal the widespread presence of m^6^A across both male and female tissues. A transcriptome-wide m^6^A map in *Anopheles gambiae* identified numerous conserved methylation sites and sex-biased differences in m^6^A regulatory gene expression. For instance, some m^6^A writer and reader components showed differential expression in reproductive tissues between sexes, although a direct role in sex determination has not yet been demonstrated (Liu et al., 2022). These findings underscore the necessity for future functional studies into the potential sex-dimorphic roles of m^6^A in non-drosophilid dipterans.

## 5 Environment and the potential link of m^6^A in sex determination

For sex determination modes influenced by environmental factors, m^6^A represents a potential bridge connecting environmental signals to gene expression. For example, in honeybee (*Ap*. *mellifera*) larvae, nutritional inputs regulate caste differentiation, and recent analyses of transcriptomic data have suggested that epitranscriptomic marks, including m^6^A, may be involved in this nutritional reprogramming (Bataglia et al., 2025). Specifically, Royal Jelly consumption activates nutrient-sensing pathways, including the target of rapamycin (mTOR) signaling cascade, which in turn modulates the expression or activity of m^6^A methyltransferases. This leads to altered m^6^A deposition on specific mRNAs, ultimately shaping epitranscriptomic profiles that drive caste-specific developmental trajectories (Bataglia et al., 2025).

Although the functional roles of these predicted m^6^A sites require experimental validation, their association with caste-biased transcripts supports a potential regulatory link between m^6^A and environmentally induced developmental fate. Similarly, hormonal signals might exert effects through modulating m^6^A. In *B*. *mori*, recent studies demonstrate that juvenile hormone (JH) analogs can regulate silk protein gene expression by altering m^6^A methylation levels on target mRNAs (Liu et al., 2023). Specifically, JH treatment modulates the expression of METTL3 and other m^6^A enzymes, thereby influencing downstream developmental processes such as silk gland differentiation. Liu et al. (2023) identified 16 silk-associated genes whose m^6^A modification and expression levels were significantly regulated by JH in the posterior silk gland, including *sericin2*, *seroin1*, *BmSPI4*, *BmSPI5*, and *Ldb*. Among these, the expression of *BmSPI4* and *BmSPI5* was directly validated to be regulated by JH through m^6^A modification of their coding sequence (CDS) regions. Furthermore, knockdown of *METTL3* affected the expression of 11 silk-associated genes, with *BmSPI4*, *BmSPI5*, *sericin2*, *seroin1*, and *Ldb* showing consistent m^6^A-mediated regulation.

**FIGURE 3:**
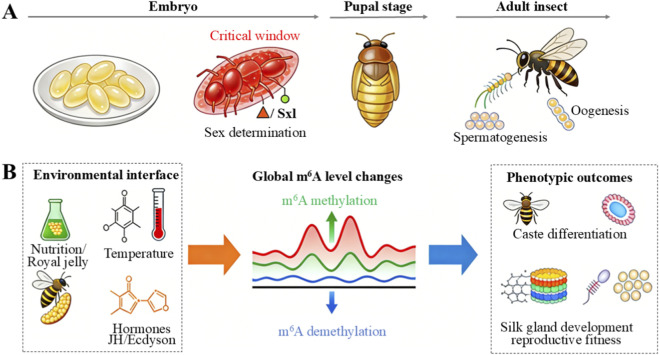
Spatiotemporal dynamics and environmental interface of m^6^A in insects. **(A)** A developmental timeline highlighting time-specific functions of m^6^A. Key windows include early embryogenesis for sex determination, such as in *Drosophila*, the pupal stage for metamorphosis, and the adult stage for gametogenesis. **(B)** Model illustrating m^6^A as a molecular bridge linking environmental inputs to phenotypic outcomes. Environmental cues such as nutrition, temperature, and hormones influence global m^6^A levels, which in turn modulate developmental processes including caste differentiation, silk gland development, and reproduction.

While direct links between temperature-dependent sex determination (TSD) and m^6^A have not been reported in insects, epigenetic marks including RNA methylation have been shown to respond to thermal stress in ectothermic vertebrates (Pu et al., 2025). These observations raise the possibility that in temperature-sensitive insect systems, m^6^A might similarly mediate temperature-induced shifts in gene expression related to sex or developmental fate (Figure 3B). Additionally, feminization or thelytoky induced by endosymbiotic bacteria—such as *Wolbachia*—may involve post-transcriptional regulation in the host, though the role of m^6^A in these processes remains unconfirmed and requires further study. Overall, the concept that environmental factors modulate sex-related gene expression via m^6^A is an emerging research frontier, with initial case-specific support (Liu et al., 2023; Bataglia et al., 2025), but lacking generalized mechanistic insight.

Several gaps limit our understanding of environment-m^6^A links. The molecular transduction from nutritional signals to m^6^A methyltransferase activity remains unknown. Whether temperature-dependent sex determination involves m^6^A, as suggested in vertebrates, is purely speculative. The potential role of m^6^A in *Wolbachia*-induced sex manipulation has not been explored. Current evidence relies heavily on correlative profiling, lacking direct experimental manipulation under controlled conditions. Advances in m^6^A editing tools are needed to establish causality.

## 6 Spatiotemporal dynamics of m^6^A modification in sex determination

m^6^A modification exhibits dynamic spatiotemporal distribution during insect sex determination processes. Along the developmental time axis, insects show changes in m^6^A levels or patterns across different stages. In *Drosophila*, for instance, the critical window for sex determination occurs within the first few hours of embryogenesis, during which correct m^6^A marking and reading are particularly crucial for the splicing of genes like *Sxl* (Kan et al., 2017). Once this window passes and the sex-specific pathways are established in male and female embryos, the direct role of m^6^A in sex determination may diminish, but it likely assumes new functions during gonad and germ cell development (Figure 3A). Studies in vertebrates like chickens have observed differences in m^6^A modification quantity between male and female embryonic gonads, suggesting m^6^A may drive distinct gene expression programs during gonad differentiation. For insects, direct comparisons of m^6^A in gonadal tissues are still limited. However, research in *Anopheles* mosquitoes found that m^6^A enrichment participates in sperm tail development during male germ cell formation (Liu et al., 2022), indicating that m^6^A retains sex-specific functional roles even during post-determination reproductive development stages.

Spatially, differences in m^6^A levels across insect tissues also hint at functional compartmentalization. In *Drosophila*, the nervous system exhibits significantly higher m^6^A levels than other tissues, consistent with its roles in behavior and memory regulation (Zhong et al., 2018). Similarly, studies in honey bees show that m^6^A modification in the brain is dynamic, with distinct patterns between young nurse bees and older forager bees, suggesting m^6^A dynamics participate in regulating the molecular basis of complex social behaviors (Bataglia et al., 2022). Although these findings do not directly address sex determination, they provide a rationale: in tissues exhibiting significant sexual dimorphism—such as male versus female gonads and pheromone glands—m^6^A modification may change across developmental stages, thereby influencing sex-specific functions. Revealing these dynamic changes currently requires employing time-course sampling coupled with high-throughput sequencing, and potentially single-cell level analysis, to capture the patterns of m^6^A gain and loss during cellular differentiation. For example, mRNA could be extracted from insect embryos at multiple time points before, during, and after sexual differentiation for m^6^A sequencing, identifying modification differences between sexes and developmental stages to construct an m^6^A dynamics atlas. Such research will help answer: Which genes undergo m^6^A modification changes during the critical sex determination period? Are these changes causative or consequential? Preliminary findings from silkworms and beetles already show that the expression and function of m^6^A machinery components are not static across embryonic and post-embryonic stages (Li et al., 2019), laying the groundwork for deeper exploration of their spatiotemporal regulatory dynamics.

Understanding spatiotemporal m^6^A dynamics faces methodological challenges. Bulk tissue analyses obscure cell-type-specific patterns critical during narrow developmental windows. Single-cell m^6^A-seq holds promise but is technically challenging and not yet applied to insect sex determination. Distinguishing causative m^6^A changes from those consequential to differentiation requires precise temporal control of m^6^A perturbation, which existing tools cannot achieve.

Beyond *Drosophila*, accumulating evidence suggests that m^6^A enrichment in the brain may be a conserved feature across insects, although systematic tissue comparisons remain limited. In the migratory locust *Locusta migratoria*, METTL3 and METTL14 are dominantly expressed in the brain and respond dynamically to crowding or isolation, with knockdown of these methyltransferases altering aggregation behaviors (Hu et al., 2025). In honey bees (*Ap*. *mellifera*), m^6^A levels in the brain differ significantly between young nurse bees and older forager bees, correlating with behavioral transitions (Bataglia et al., 2022), and m^6^A machinery genes show brain-specific expression patterns (Bataglia et al., 2022). Studies in ants (*Solenopsis invicta*) have directly demonstrated m^6^A modification of dopamine receptor and transporter mRNAs in the brain, where manipulation of m^6^A levels shifts worker behavior between foraging and nursing roles (Chen et al., 2024). While direct m^6^A mapping data in brain tissues are not yet available for beetles (*Tribolium*) or silkworm (*Bombyx*), the consistent association between m^6^A and neural function across Orthoptera, Hymenoptera, and Diptera suggests that brain enrichment of m^6^A may represent an ancestral feature that has been co-opted for lineage-specific behavioral and developmental regulations. This tissue-specific pattern further supports the model that m^6^A functions are not monolithic but are deployed in distinct tissues to serve diverse biological roles—from sex determination in *Drosophila* brains to caste-specific behavior in social insects.

## 7 Similarities and differences in m^6^A-mediated sex regulation across insect taxa

Comparative analysis across insect taxa reveals a fundamental evolutionary pattern: while the core m^6^A methylation machinery is broadly conserved, its functional integration with sex determination pathways is highly lineage-specific and contingent upon the underlying genetic architecture of sex determination.

In dipterans such as *D*. *melanogaster*, which employ an *Sxl*-centric sex determination cascade, m^6^A has been co-opted as an essential regulatory layer ensuring female-specific splicing of *Sxl* and subsequent female development (Haussmann et al., 2016; Lence et al., 2016). This tight integration is exemplified by the fact that classical female-lethal genes *Fl(2)d* and *Virilizer* were later identified as core components of the m^6^A methyltransferase complex (Granadino et al., 1990; Hilfiker et al., 1995). However, even within Diptera, this mechanism is not universal; in mosquitoes such as *An*. *sinensis*, which lack an *Sxl*-dependent pathway, m6A plays no apparent role in primary sex determination but is instead involved in spermatogenesis (Liu et al., 2022).

In insect orders that do not utilize *Sxl* as a sex determination switch—including Coleoptera (*T*. *castaneum*), Hymenoptera (*Apis mellifera*), and Lepidoptera (*B*. *mori*)—m^6^A depletion does not disrupt core sex differentiation. Instead, m^6^A serves broader developmental functions: metamorphosis and gametogenesis in *Tribolium* (Jiao and Palli, 2023), nutrition-mediated caste differentiation in *honey bees*(Wang et al., 2021), and embryonic development and silk gland regulation in *Bombyx* (Liu et al., 2023; Li et al., 2019). These findings indicate that in the absence of an *Sxl*-dependent mechanism, m^6^A has been repurposed to support diverse post-embryonic and reproductive processes.

This functional divergence underscores the evolutionary plasticity of epitranscriptomic regulation: m^6^A has been repeatedly recruited to serve lineage-specific developmental needs, ranging from sex determination to metamorphosis, reproduction, and environmentally cued phenotypic plasticity. The conservation of the core m^6^A machinery across insects, coupled with the striking variability in its functional outputs, provides a powerful framework for understanding how epigenetic regulatory layers evolve in parallel with genetic sex determination mechanisms.

Critical gaps limit cross-species comparisons. Functional data exist for only a handful of species, with most orders uncharacterized. Inconsistent experimental approaches (RNAi efficiency, developmental stages) complicate comparisons, as exemplified by conflicting *honey bee* findings. The molecular basis of m^6^A target divergence between *Sxl*-dependent and -independent species is unexplored. m^6^A roles in environmental sex determination or paternal genome elimination have not been investigated. Systematic, methodologically consistent surveys across broader phylogeny are needed.

Future studies should prioritize cross-taxon comparisons of m^6^A mutant phenotypes and the identification of taxon-specific m^6^A target transcripts to further elucidate how m^6^A functions have diversified alongside the evolution of sex determination strategies. A straightforward yet powerful hypothesis emerging from the observed species-specific functions is that differential mRNA targeting by the m^6^A methyltransferase complex may underlie much of this diversification. In this view, the core catalytic machinery is conserved, but the suite of transcripts it modifies—and thus the biological processes it influences—varies across species depending on how the complex is recruited to specific DRACH sites (Pandey et al., 2025). This hypothesis directly engages a central unanswered question in the epitranscriptomics field: what determines which DRACH sites are methylated in a given cell type or species? Potential mechanisms include variation in the expression or specificity of auxiliary factors that recruit the complex to particular transcripts, divergence in cis-regulatory elements flanking DRACH sites, or species-specific interactions with RNA-binding proteins that guide the writer complex. While differential targeting is an attractive explanation, it is unlikely to account for all observed differences. Other contributing factors may include lineage-specific evolution of downstream pathways, such as loss of Sxl in non-Drosophilids (Ruiz et al., 2013), differential requirements for m^6^A in tissue-specific processes (Li et al., 2014), or functional redundancy among m^6^A targets that buffers against phenotypic change in some species (Lasman et al., 2020). Testing these possibilities will require systematic comparative m^6^A profiling across multiple insect orders under standardized conditions, coupled with functional validation of species-specific targets through cross-species rescue experiments. Such approaches could reveal whether the m^6^A machinery has been repeatedly co-opted to regulate different developmental processes by simply shifting its target repertoire, or whether more fundamental changes in the regulatory logic of the pathway have occurred.

## 8 Conclusion

In conclusion, m^6^A methylation plays an important and distinctive role in insect sex determination and development, with functions ranging from a critical switch in *Drosophila* to broader roles in metamorphosis, reproduction, and caste differentiation in other insects (Haussmann et al., 2016; Jiao and Palli, 2023; Wang et al., 2021). This diversity reflects the flexible evolutionary utilization of epitranscriptomic regulation across insects (Maleszka, 2016).

Several key questions emerge from this synthesis, defining a roadmap for future research. First, resolving the spatiotemporal dynamics of m^6^A at cellular resolution is paramount. The application of emerging techniques like single-cell m^6^A-seq and spatial transcriptomics will be crucial to identify the specific cell populations where m^6^A exerts its regulatory influence during critical developmental windows.

Second, the molecular mechanisms underlying m^6^A function demand deeper investigation. This includes dissecting the differential requirements for catalytic versus auxiliary subunits, for example, why mutations in *Fl(2)d* and *Virilizer* cause more severe phenotypes than loss of METTL3 in *Drosophila*? and elucidating the precise mode of action of reader proteins like YT521-B in splicing regulation. Furthermore, understanding how environmental cues such as nutrition, temperature, and endocrine signals are transduced into epitranscriptomic changes is critical to establish m^6^A as a molecular bridge linking environment to developmental plasticity.

Third, a broader comparative framework is essential. Expanding taxonomic coverage beyond traditional models to include understudied orders, such as Hemiptera and Orthoptera, will test the generality of current models. A central, unresolved question is whether the lineage-specific functions of m^6^A arise primarily from differential mRNA targeting by the methyltransferase complex. Systematic, methodologically consistent cross-species m^6^A profiling, coupled with functional validation of species-specific targets, is needed to explore this hypothesis and understand how m^6^A functions have diversified alongside sex determination strategies.

Finally, from an applied perspective, harnessing m^6^A regulatory mechanisms offers promising strategies for insect management, from disrupting reproduction in agricultural pests to supporting colony health in beneficial pollinators.
